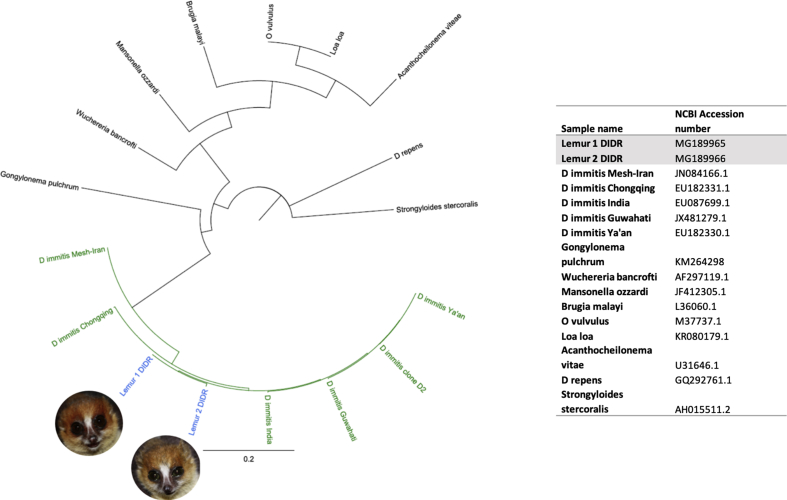# Causative agent of canine heartworm (*Dirofilaria immitis*) detected in wild lemurs

**DOI:** 10.1016/j.ijppaw.2019.04.005

**Published:** 2019-04-14

**Authors:** Sarah Zohdy, Kim Valenta, Bernadette Rabaoarivola, Caitlin J. Karanewsky, Weam Zaky, Nils Pilotte, Steven A. Williams, Colin A. Chapman, Zach J. Farris

**Affiliations:** a School of Forestry and Wildlife Sciences, Auburn University, Auburn, AL, 36849, USA; b College of Veterinary Medicine, Auburn University, Auburn, AL, 36849, USA; c Department of Evolutionary Anthropology, Duke University, Durham, NC, 27708, USA; d Centre ValBio, Ranomafana, Madagascar; e Department of Biochemistry, Stanford University School of Medicine, California, USA; f Department of Biological Sciences, Smith College, Northampton, MA, USA; g Molecular and Cellular Biology Program, University of Massachusetts, Amherst, MA, USA; h Department of Anthropology, McGill University, Montreal, Quebec, Canada; i Wildlife Conservation Society, 2300 Southern Boulevard, Bronx, NY, USA; j Section of Social Systems Evolution, Primate Research Institute, Kyoto University, Japan; k Department of Health & Exercise Science, Appalachian State University, North Carolina, USA

**Keywords:** Madagascar conservation, Pathogen spillover, Mosquito-borne disease, Blood parasite, Canine vector-borne diseases, brown mouse lemur, *Microcebus rufus*

## Abstract

The lemurs of Madagascar are threatened by human activities. We present the first molecular detection of canine heartworm (*Dirofilaria immitis*) in a wild non-human primate, the mouse lemur (*Microcebus rufus*). Zoonotic *D. immitis* infection has been associated with clinical pathology that includes serious and often fatal cardiac and pulmonary reactions. With human encroachment and associated increases in free-roaming dog populations in Madagascar, we examined lemurs for zoonotic canid pathogens. *D. immitis* presents a new potential conservation threat to lemurs. We highlight the need for wide-ranging and effective interventions, particularly near protected areas, to address this growing conservation issue.

## Highlights

• Molecular evidence of canine heartworm (*Dirofilaria immitis*) in lemurs.
• The first detection of *D. immitis*, in a wild non-human primate.
• Free-roaming dogs spatially overlap with lemurs.

## Graphical abstract

**Figure:**
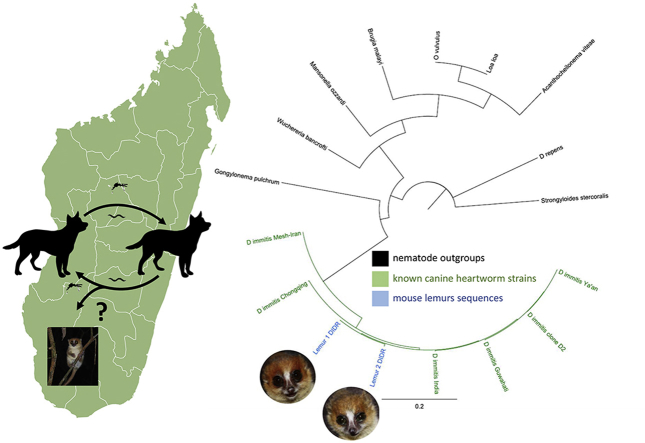

## 1 Introduction

The five lemur families are amongst the world's most imperiled groups of vertebrates with at least 94% of 101 species identified as threatened by the International Union for the Conservation of Nature (Schwitzer et al., 2013). Endemic to the biodiversity hotspot of Madagascar, lemur diversity is particularly remarkable considering that Madagascar represents only a small fraction of tropical landmass (Kremen et al., 2008). Threats to lemurs primarily include habitat loss and bushmeat hunting (Barrett, 2010; Borgerson et al., 2016; Brooks et al., 2002) which result from non-sustainable land use and resource extraction driven by poverty and a legacy of political instability (Waeber et al., 2016). Today the forests of Madagascar cover 92,200 km^2^ (approximately the size of Portugal), approximately 10% of the original forested area. Between 2000 and 2010 the country lost 9,700 km^2^ of forest, which is almost three times the area of loss in the previous decade (Schwitzer et al., 2014; Doherty et al., 2016; Kim et al., 2015).

Introduced predators also have the potential to devastate lemur populations, but this has not received sufficient research attention (Doherty et al., 2016). Globally, free-ranging domestic dogs affect wildlife through predation, competition, hybridization, and disease transmission (Young et al., 2011; Koster and Noss, 2014; Vanak and Gompper, 2010; Leonard et al., 2014; Rasambainarivo et al., 2017; Hughes and Macdonald, 2013). To date, little attention has been paid to the threat of dogs as disease vectors. In Madagascar, free-roaming dog populations have been shown to have negative effects on lemur populations (Farris et al., 2014) and numerous other endemic wildlife species (Farris et al., 2015a, 2015b); however, pathogen transfer from dogs to native wildlife has not yet been investigated (Rasambainarivo et al., 2017). Here, we investigate the potential for pathogen (*Dirofilaria* immitis) spillover from dogs to wild mouse lemurs (*Microcebus rufus*), non-human primates with known co-occupancy with dogs (Farris et al., 2014).

## 2 Materials and methods

Blood samples were collected from 47 mouse lemurs in Ranomafana National Park (RNP), Madagascar, where free-roaming dog populations are high (occupancy estimated at 0.78 ± SE 0.08) (Farris et al., 2017). Mouse lemurs were caught in live traps, given a thorough physical health examination, and 5 μL of blood was collected on TropBio Dried Blood Spot cards (Cellabs LTY, Australia) (IACUC #27439 and #20162897). Spot cards were protected in a sterile, sealed container with desiccant and left to dry overnight. In addition, freshly collected blood was also used to generate thick (10 μL) and thin (5 μL) blood smears which were prepared using a Giemsa stain.

In addition to the collection of blood from mouse lemurs, 5 μL of whole blood was also collected from 18 dogs living in and around RNP during a spay-neuter-vaccination campaign for community dogs. As described above, these samples were collected on TropBio Blood Spot cards.

All samples (along with negative controls) were extracted using the Qiagen DNEasy Blood and Tissue Kit (Qiagen, Hilden, Germany) and screened for the presence of filarial parasites using a “pan-filarial” primer set (Rishniw et al., 2006) designed to produce an amplicon from filarial worms through the amplification of a target sequence spanning a segment of the 5.8S-ITS2-28S ribosomal region (Table 1). PCR Master mix was made by combining 11 μL of nuclease-free H_2_O, 5 μl of 5× Phire Reaction buffer, 0.5 μL dNTPs (10 μM), 1 μL of each primer, 0.5 μL of Phire Enzyme, and 1 μL of DNA template, for a total volume of 20 μL. Thermal cycling conditions were 95 °C for 30 s, then 35 cycles of denaturing (30 s at 95 °C), annealing (40 s at 55 °C) and extension (1 min at 72 °C); a final extension (5 min at 72 °C) and a hold at 4 °C in a Veriti 96 well Thermal Cycler (Applied Biosystems, Inc., Foster City, CA). PCR products were then run on a 1.5% agarose gel and visualized for the presence of bands. The expected size of an amplicon resulting from the amplification of a *D. immitis* target was 542 bp and two samples produced distinct bands appearing to be approximately this size. These samples were subjected to cycle sequencing, using 4 μL of Big Dye, 2 μL of primers (0.8 pMol/μL), and 4 μL of PCR product. Two sequencing reactions were prepared for each positive sample (i.e. those giving a band appearing to be 542 bp) using forward and reverse primers. The cycling protocol was set at 25 total cycles of 96 °C for 30 s, 50 °C for 15 s, and 60 °C for 4 min. Reaction products were purified using EdgeBio columns (EdgeBiosystem Inc., Gaithersburg, MD) according to the manufacturer's protocol. Purified products were then sequenced using an ABI 3130xl Genetic Analyzer (Applied Biosystems) and resulting sequence reads underwent BLAST analysis. BLAST results indicated the unambiguous presence of *D. immitis* in both analyzed samples (Sample 1: Best match to NCBI Accession JX866681.1; 100% query coverage; 98% identity; E value = 0.0. Sample 2: Best match to NCBI Accession KY863453.1; 100% query coverage; 99% identity, E value = 0.0).

**Table 1:** Primer sequences for nested PCR designed specifically for *D. immitis* detection.

Primer Pairs	Primer Orientation	Primer Sequence
“Pan Filarial” PCR	5.8S-ITS2-28S	5′-AGT GCG AAT TGC AGA CGC ATT GAG-3′
“Pan Filarial” PCR	5.8S-ITS2-28S	5′-AGC GGG TAA TCA CGA CTG AGT TGA-3′
Primary 5s PCR (5S-rDNA)	Forward	5′-GTTAAGCAACGTTGGGCCTGG-3′
	Reverse	5′-TTGACAGATCGGACGAGATG-3′
Nested 5S-sp PCR (5S spacer)	Forward	5′-CAAGCCATTTTTCGATGCACT-3′
	Reverse	5′-CCATTGTACCGCTTACTACTC-3′

For verification, *D. immitis* positive samples were also amplified using a *D. immitis*-specific nested PCR detection protocol (Lizotte-Waniewski and Williams, 2001) with primers targeting the 5S-rDNA gene and nested primers targeting the spacer region within this gene (Table 1). Both initial PCR reactions, and nested reactions were performed in 50 μL reaction volumes, utilizing reaction recipes and cycling protocols described in (Lizotte-Waniewski and Williams, 2001).

## 3 Results and discussion

Sequencing of the purified “pan-filarial” PCR products coupled with BLAST analyses of these results confirmed the presence of *D. immitis* DNA in both samples found to be positive using the “pan-filarial” primer set described above. Nested PCR analysis followed by sequencing provided further confirmation of positivity in one of the two “pan-filarial” positive samples (Best match to NCBI Accession EU360965.1; 91% query coverage; 98% identity; E value = 2e-58). Unfortunately, nested PCR failed to produce sequence-quality product from the other “pan-filarial” positive sample, at which point extracted DNA was exhausted, preventing further analysis. However, the lack of sequencing results from the nested PCR analysis of the second sample does not preclude the detection of *D. immitis* confirmed by sequencing of the “pan-filarial” assay products. When compared in GenBank to other known *Dirofilaria* spp. and other nematode outgroups, our samples matched specifically to *D. immitis* (Supplementary Table 1). Infected individuals did not appear to have any respiratory or cardiac abnormalities, or any other diagnostically relevant symptoms.

In addition to the detection of *D. immitis* in the lemur samples, seven of the dogs sampled from RNP produced gel product bands when tested using the “pan-filarial” assay described above. Three of these samples produced sequences which were closely matched to *D. immitis.* Historic records in Madagascar also suggested the presence of *D. immitis* in dogs, in-country (Daynes, 1964).

To investigate the underlying cause of the sequencing positivity in lemurs, thin and thick blood smears were made for morphological parasite identification. The lemurs found to be positive using “pan-filarial” primers both had microfilariae in their blood smears; however, these could not be confirmed as *D. immitis* based on morphology due to long-term slide storage conditions. It is possible that the molecular detection of *D. immitis* DNA in lemurs may be the result of pre-patent infection of infective larvae from a very recent mosquito bite. Our findings could also be due to the presence of larva migrans, or larvae that do not mature into adults, as is often seen in the organs of other non-definitive hosts like felids and humans. However, given that *D. immitis* was detected from blood samples and not organ tissues this is not likely.

## 4 Conclusions

Here we provide molecular evidence of a possible pathogen threat to lemur populations from domestic dogs. *D. immitis*, the mosquito-borne causative agent of canine heartworm has not previously been confirmed in non-human primates, although filaroid DNA has been detected in another lemur species (Springer et al., 2015). Cases of *D. immitis* infection have been identified in a small number of wild species more closely related to dogs, including ferrets (*Mustela putorius*), sea lions (*Zalophus californianus*), beavers (*Castor canadensis* (Foil and Orihel, 1975), and raccoon dogs (*Nyctereutes procyonoides*), (Kido et al., 2011). Clinical effects of *D. immitis* in non-definitive hosts include serious and often fatal cardiac and pulmonary reactions (McCall et al., 2008; Litster and Atwell, 2008), although host species and body size may influence clinical outcome. *D. immitis* has been documented in humans, where clinical pathology of infection includes coin lesions on the lungs, ocular infections, and cardiomyopathy (Otto, 1975; Lee et al., 2010). Since clinical manifestations of infection in these non-definitive taxa are very damaging, this would likely be the case in lemurs, if patent infection did occur. However, for the two animals found to have *D. immitis* in this study, we did not detect clinical signs of pathology.

Pathogen spillover events from invasive species are an underappreciated threat to the beleaguered island fauna of Madagascar, and will likely increase with increasing encroachment into what remains of Madagascar's forests. Our findings add pathogens from dogs to the list of potential threats facing lemurs. The unambiguous presence of *D. immitis* DNA in two samples of mouse lemur blood may indicate the presence of microfilariae in this host species and suggests that adult worms are active and breeding in mouse lemurs; however, given that no cases of patent *D. immitis* infections are known in primates or other aberrant hosts, this is unlikely. The detection of *D. immitis* DNA in a region where dogs are also positive for *D. immitis* does suggest contact between a mosquito vector infected with canine heartworm and wild mouse lemurs, highlighting the potential for exposure of native wildlife to canid pathogens from invasive carnivores. Mouse lemurs could be a sentinel species of emerging zoonotic infections in Madagascar due to their broad habitat usage and the detection of many pathogens in these populations (Zohdy et al., 2015; Raharivololona and Ganzhorn, 2009; Bublitz et al., 2015).

## Figures and Tables

**figs1:**